# Subsistence Food Production Practices: An Approach to Food Security and Good Health

**DOI:** 10.3390/ijerph14101184

**Published:** 2017-10-05

**Authors:** Sejabaledi A. Rankoana

**Affiliations:** Department of Sociology and Anthropology, University of Limpopo, Private Bag x 1106, Sovenga 0727, South Africa; sejabaledi.rankoana@ul.ac.za; Tel.: +27-(0)-152682179 or +27-(0)-724431321

**Keywords:** self-sufficiency, traditional foods, subsistence production, food security, health food, maternal care

## Abstract

Food security is a prerequisite for health. Availability and accessibility of food in rural areas is mainly achieved through subsistence production in which community members use local practices to produce and preserve food. Subsistence food production ensures self-sufficiency and reduction of poverty and hunger. The main emphasis with the present study is examining subsistence farming and collection of edible plant materials to fulfill dietary requirements, thereby ensuring food security and good health. Data collected from a purposive sample show that subsistence crops produced in the home-gardens and fields, and those collected from the wild, are sources of grain, vegetables and legumes. Sources of grain and legumes are produced in the home-gardens and fields, whereas vegetables sources are mostly collected in the wild and fewer in the home-gardens. These food sources have perceived health potential in child and maternal care of primary health care.

## 1. Introduction

Food security is a prerequisite for health [1], which is achieved when people have consistent physical and economic access to sufficient, safe and nutritious foods to meet their dietary needs and food preferences. Safe and nutritious foods are procured through subsistence production practices adopted by particular human cultures [2]. The most common food production practices include subsistence farming, gathering, hunting and fishing, which are key drivers to human health and well-being, and have remained the main sources of livelihood for majority of the rural population in Africa [3]. Rural community members produce and preserve foods to end poverty and malnutrition, and ensure self-sufficiency [4,5,6]. Pitso and Lebese [7] corroborate that reliance on traditional foods provides a viable alternative to government food security strategies in Southern Africa [8]. Dlamini et al. [9] add that South Africa’s traditional foods and native knowledge of food production could potentially be used to identify concepts and products for different markets, such as edible plants as sources of new and natural colourants and flavourants, and nutritional supplements to control hunger. For Webb and Von Braun [10], these foods are consumed to cope with food insecurity. In Ethiopia, for example; more than 95% of households supplement diet with famine foods, such as roots, leaves and grass [11]. Continued production and dependency on subsistence foods is part of cultural heritage that ensures food availability and accessibility to end poverty and malnutrition [12,13]. This paper is therefore, intended to describe the subsistence practices of food production with emphasis on subsistence farming and collection of edible plant materials to fulfill dietary requirements, thereby ensuring food security and good health.

## 2. Materials and Methods

A qualitative study was carried out among the Northern Sotho of Sekhukhune District Municipality of Limpopo Province, South Africa. The municipality is rural and covers an area of 13,528 km^2^ with a population of about 1,169,762. The average household size is 4.7 with 51.2% female-headed households. Unemployment rate is at 50.9% while youth joblessness is at 60.6%. The people still produce subsistence food crops in the home-gardens [14]. Cultural heritage and indigenous knowledge systems are sustained by practices such as rites of passage and production of food crops [15]. Purposive sampling was used to make up the study sample in Mohlaletsi community. The community falls within Sekhukhune District. Since the study interest is on subsistence food production practices, only heads of households aged above 25 years were purposively selected in the community. Semi-structured interviews were conducted with 120 heads of households. They were 76 males and 44 females. Face-to-face interviews were conducted with participants. The main questions asked during the interviews included description of subsistence farming and collection practices of procuring foods. Data were analysed by means of thematic content, which produced data that show subsistence farming and gathering of edible plant materials as the main practices of securing food to ensure self-sufficiency and avoid poverty and malnutrition. In the study, sources of grain and legumes (five crops) are produced in the home-gardens and ploughing fields. Of the 11 vegetable sources, four crops are cultivated in the home-gardens and fields, while seven grow as weeds spontaneously in the home-gardens, fields and the wild. During data collection process, subsistence farming and collection practices were fully described by participants. Cultivated and collected food crops are presented in terms of scientific and family names obtained from the University of Limpopo Herbarium database of subsistence food plants of Limpopo Province. The voucher specimens and files of the crops identified by the participants were selected. The crops’ scientific, family and vernacular names, description of use and frequency of identification are presented in Table 1, Table 2 and Table 3 and categorised according to food sources as grain, legumes and vegetables. Ethical approval was obtained from University of Limpopo Ethics Committee for a master’s project on Ethnobotany of a rural community in Limpopo Province (TREC/28/2013: PG).

## 3. Results and Discussion

### 3.1. Subsistence Food Production Practices

#### 3.1.1. Subsistence Farming

In the study, food crops are cultivated with hand-hoes in the home-gardens or by a tractor at a cost of R1200.00 ($89.55) per hector in the fields. Planting commences after the first rain, usually between October and December. Manuring of the soil is not a common practice, though cattle or poultry manure is sometimes used. All the crops are grown together in the same field. The seeds are mixed and sown simultaneously. The most common crops cultivated are *leotša* (*Pennisetum picatum*) and *lebelethoro* (*Andropogon sorghum*)*,* and *lefela* (*Zea mays*) which are sources of grain. Participants (86%) reported that as the young crops shoot up, the accompanying weeds are removed with a hand hoe. The agricultural season ends around May to mid-June with the final harvest when the ears of *Pennisetum picatum and Andropogon sorghum and Zea mays* are broken off by hand. These are threshed with wooden flails on a specially prepared floor of hard beaten soil known as *segotlo* (backyard). *Lefela* may be threshed in the same way on a different floor, but more often it is husked and shelled by hand. The average amount of harvest is five bags of grain of either of the crops. The stalks are left standing as feed for livestock. Legumes (*Vigna sinensis* and *Voandzeia subterranean*) are generally gathered about the same time. The estimated quantity of fresh legumes harvested for household consumption is dependable on the family size, which ranges from 2 kg for a family of 2–4 members, and more than 2 kg for a family of about 5–8 members. Grain obtained from mature *Sorghum, Pennisetum* and *Zea mays* is a source of meal used to prepare a hard porridge, which is the staple food of the community. Meal is obtained by pounding grain with a mortar and pestle or grounded with grinding stones. All grain which is not immediately needed is stored and preserved by mixing it with ash resulting from burnt leaves of *sekgophana* (*Aloe marlothii*) to protect it against attack by weevils. The seeds could be stored for up to three years. Another preservation method reported is sun-drying of vegetable materials for future use, in which fresh, tender leaves of all vegetable sources are put in the sun to dry. Sometimes the cooked materials are spread on the steel-sheets and put in the sun to dry. The dried materials are stored in plastic bags and buckets for future use. Information about the nutritional value of the crops presented in Table 1, Table 2 and Table 3 is derived from Nell and Siebrits [16], Murovhi and Materechera [17] and Faber et al. [18].

Additionally, the traditional varieties of legumes; *monawa* (fruit of *Vigna sinensis*) and *tloo-marapo* (fruit of *Vosndzeia subterraneana*) are cultivated as sources of protein.

In addition to sources of grain and legumes, participants also mentioned sources of vegetable such as *lerotse* (the fruit of *Citrullus vulgaris*) and *lefodi* (the fruit of *Cucurbita pepo*) and *leraka* (fruit of *Lagenaria vulgaris*) produced in the home-gardens and fields. The crops are presented in Table 3 with other vegetable sources.

Ojelel and Kakudidi [6] attest that majority of South African rural communities procure health foods through subsistence farming [19,20]. The World Bank Report [21] and The Rockefeller Foundation [22] note that subsistence farming is important for food security for the majority of the rural poor. Musinguzi et al. [23] observe that majority of traditional foods are consumed to avoid food insecurity and widespread poverty in African countries, which are inextricably linked to low agricultural productivity aggravated by climate variability. Despite the negative impact of climate change on food security, rural communities have the capacity to produce food through their traditional systems for sustainable livelihood [24,25,26].

#### 3.1.2. Collection of Edible Plant Materials

*Theepe* (Amaranthus thunbergii), *monyaku* (*Cucumis africanus*), *lerotho* (*Cleome monophylla*), *letelele (Amaranthus spinosus), lehlanye (Vernonia fastigiata), mošitšana (Elephantorriza elephantine)* and *tshehlo (Tribulus terrestris)* presented in Table 3, were identified as the main vegetable sources collected in the wild and home-gardens. 

These crops grow as weeds between crops in the home-gardens and ploughing fields. The crops appear after the first rain between October and March, and are harvested and used directly without any form of trade. Fresh tender leaves are collected, washed, boiled and seasoned with salt to make delicious and popular local vegetables. Very often Elephantorriza elephantine and Amaranthus are cooked with Cleome monophylla and Cucumis africanus to reduce their mild bitter taste. These observations are supported by Faber et al. [18], Rampedi and Oliver [27], and Baiphethi and Jacobs [28] that South Africa has a wealth of local flora most of which provides poor households with leafy vegetables to ensure local food security. Chadare et al. [29] add that subsistence food sources collected from the wild augment and diversify diet, thereby ensuring good health and well-being. Shumsky et al. [30] corroborate these results that in the semi-arid communities of Kenya, wild vegetables are consumed to end hunger. Vegetable crops are the largest group of species consumed in Bulgaria, with above-ground parts; young leaves, shoots and stems gathered and used as vegetables [31]. Kwenin et al. [32] believe that under-utilisation of these food sources may pose threat to food security as they are important commodities for poor households’ food security to ensure good health and well-being.

### 3.2. Health Potential of Subsistence Foods

In the study, food produced through subsistence farming and collection in the wild, is consumed to ensure household food availability. Participants (78%) reported that they produce and consume the food to avoid lack of enough food to maintain their families. Unemployed household heads (23 males, 16 females) reported that they ensure constant production of crops to obtain meal to cook porridge, and legumes and vegetables to relish porridge to ensure food availability in the households. Of the participants, 83% reported that food produced from subsistence crops is nutritious in that it is uncommon for one to become allergic to the porridge, whole-grain stew and vegetables. Participants (56%) maintained that usually they feel healthy and well after they had eaten subsistence foods. The food is usually prescribed for family members as a preventive and curative measure. For example, a child of between 6 and 9 months is fed with sorghum porridge for normal growth and health. This food prevents susceptibility to potential childhood developmental diseases such as kwashiorkor and marasmus caused by malnutrition. Adults consume the foods to obtain a balanced diet (starch, vegetables and legumes) to ensure food availability. Sorghum and millet porridge relished by a vegetable prepared from *Amaranthus* species is helpful in easing child labour (because of its slimy texture), maintaining a normal menstruation cycle and control of persistent constipation. Good health is maintained through exercise when people engage in labour to produce subsistence crops and frequent expeditions to collect vegetables in the wild.

The nutritional value of *Cleome monophylla* and *Amaranthus* species identified by participants forms a requisite part of the daily primary nutrition of many South Africans [33]. The vegetables are identified by Faber et al. [18] as sources of vitamins A and C, and folate, whose low intake is among the top ten risk factors contributing to child developmental diseases such as anaemia, kwashiorkor and marasmus. The health potential of *Vigna sinensis* and *Vosndzeia subterraneana* are corroborated by Felhaber [34] that beans and nuts provide the body with protein that is cheaper than meat and fish, and supplies the body with different vitamins and nutrients.

## 4. Conclusions

The objective of this study was to describe the subsistence practices of food production with emphasis on subsistence farming and collection of edible plant materials to fulfill dietary requirements, thereby ensuring food security and good health. Data were collected in a rural community among household heads aged above 25 years. The study results show that food is produced through subsistence farming and collection of edible plant materials from the wild. Of the crops produced in the home-gardens and fields, three are sources of grain, which is pounded or grounded to make meal used to prepare porridge; the staple food in the community. Protein is obtained from two legumes also cultivated in the home-gardens. Fewer vegetable sources are sourced in the home-gardens, whereas a bulk of vegetables is derived from crops that grow spontaneously in the wild. These foods are procured to ensure food availability and accessibility at household level. Most importantly, the foods have health potential for preventive and curative care of primary health care. The foods are important for maternal and child health and development. Food production practices reported in the study are specific to the culture of the Northern Sotho of Sekhukhune, and affordable means of ensuring food security and good health. Despite the negative impact of rainfall scarcity and increased temperature in the area, continued subsistence food is encouraged. This cultural practice may be useful to eradicate poverty and ensure healthy lives and well-being.

## Figures and Tables

**Table 1:** Grain sources.

Family Name	Scientific Name	Common Name	Vernacular	Description of Use	Frequency		Nutrients
					Male	Female	
Gramineae	*Andropogon sorghum* Brot.	Sorghum	*Lebelethoro*	Matured heads are harvested, threshed and grounded to make fine meal used to prepare porridge	76	44	Carbohydrates, starch and dietary fibre
	*Pennisetum spicatum* Koern & vars.	Finger-millet	*Leotša*	Matured heads are harvested, threshed and grounded to make fine meal used to prepare porridge	76	44	Contains 5 to 7% oil, and has higher protein and energy levels than maize and sorghum
	*Zea mays* L.	Maize	*Lefela*	Fresh cobs are boiled or grilled to make a snack. Dried cobs are harvested, threshed and grounded to make fine meal used to prepare porridge	76	44	Starch, protein

**Table 2:** Legume sources.

Family Name	Scientific Name	Common Name	Vernacular Name	Description of Use	Frequency		Nutrients
					Male	Female	
Leguminiceae	*Vigna sinensis* Endb.	Cow pea	*Monawa*	Fresh leaves are boiled to make relish. The pods are boiled and eaten as snack. Dried beans are harvested, threshed and prepared as side-dish or whole-grain stew	76	44	Fiber, Calcium, Carbohydrate, Iron, Crude protein
	*Voandzeia subterraneana*	Njugo bean	*Tloo-marapo*	Fresh underground tubers are harvested, boiled and eaten raw Dried beans are harvested, threshed to prepare relish or whole-grain stew	76	44	Carbohydrate, Calcium, Crude protein

**Table 3:** Vegetable sources.

Family Name	Scientific Name	Common Name	Vernacular Name	Description of Use	Frequency		Nutrients
Amaranthaceae	*Amaranthus thunbergii* Moq.	Pigweed	*Theepe*	Fresh, tender leaves are boiled to make a palatable side-dish. Dried leaves or cooked side-dish	53	47	Vitamins A & C, Calcium, Iron, Thiamine, Phosphate,
	*Amaranthus spinosus*	Thorny pigweed	*Letelele*	Fresh leaves are boiled to make slimy relish	12	47	Crude protein
Asteraceae	*Vernonia fastigiata.*	Langbeen bossie	*Lehlanye*	Tender leaves are boiled to make bitter-taste vegetable	19	47	Vitamin C, Crude protein
Capparidaceae	*Cleome monophylla* L.	Spider whips	*Lerotho*	Young leaves and flowers are boiled to make relish	53	47	Iron, Calcium, Phosphorus, Magnesium, Omega 3, 𝛽-Carotene, Folate
Cucurbitaceae	*Citrullus vulgaris* Schrad Ex. E and Z.	Bitter melon	*Morotse*	Tender leaves and immature fruits are boiled to make relish	53	47	Vitamin C, Crude protein
	*Citrullus vulgaris* Schrad. Vars.	Watermelon	*Mogapu*	Well-cooked tender leaves make palatable relish	22	47	Vitamin C
	*Cucubita pepo.* L.	Pumpkin	*Mofodi*	Immature leaves, fruits and flowers make relish	53	47	Fibre, Iron, CarbohydrateCalcium, Vitamins A & C
	*Cucumis africanus* L.f.	Wild cucumber	*Monyaku*	Tender leaves make relish	53	47	Vitamin C, Crude protein
	*Lagenaria vulgaris* ser.	Gourd	*Moraka*	Immature leaves and fruits make relish	16	47	Crude Protein, Vitamin C
Fabaceae	*Elephantorriza elephantine* Burkei Benth.	Fern plant	*Mošitšana*	Young, tender leaves make a relish	14	47	Calcium, Iron, Crude protein
Zygophyllaceae	*Tribulus terrestris.*	Devil’s thorn	*Tshehlo*	Fresh leaves make a relish	8	47	Iron, Calcium

## References

[1] Coupland R. (2007). Security, insecurity and health. Bull. World Health Organ..

[2] Kuhnlein H.V., Receveur O. (1996). Dietary change and food systems of indigenous peoples. Annu. Rev. Nutr..

[3] Food and Agriculture Organization of the United Nations (FAO) (2015). Socio-Economic Context and Role of Agriculture. Country Fact Sheet on Food and Agriculture Policy Trends.

[4] United States Agency for International Development (USAID) (2001). The Potential of Indigenous Wild Foods. Workshop Proc..

[5] Doss C.R. (2002). Men’s Crops? Women’s Crops? The Gender Patterns of Cropping in Ghana. World Dev..

[6] Ojelel S., Kakudidi E.K. (2015). Wild edible plant species utilized by a subsistence farming community in Obalanga sub-county, Amuria District, Uganda. J. Ethnobiol. Ethnomed..

[7] Pitso F.S., Lebese M.R. (2014). Traditional use of wild edible plants in arid areas of South Africa. J. Hum. Ecol..

[8] Food and Agriculture Organization of the United Nations (FAO) Developing a climate-smart agriculture strategy at the country level: Lessons from recent experience. Proceedings of the Background Paper for the Second Global Conference on Agriculture, Food Security and Climate Change.

[9] Dlamini N.R., Moroka T., Mlotshwa L., Reddy J., Botha G. (2010). Indigenous edible plants as sources of nutrients and health benefiting components (nutraceuticals). Science Real and Relevance Conference.

[10] Webb P., Von Braun J. (1994). Famine and Food Security in Ethiopia. Lessons for Africa.

[11] Guinand Y., Dechassa L. (2000). Wild-Food Plants in Southern Ethiopia: Reflections on the Role of Famine-Foods at a Time of Drought.

[12] Brooks S., Loevinsohn M. Shaping Agricultural Innovation Systems Responsive to Food Insecurity and Climate Change. http://eprints.whiterose.ac.uk/90766/1/SBnrf5aug2011.pdf.

[13] Tafesse A., Ayele G., Ketema M., Geta E. (2015). Food security and adaptation strategies to climate change in Eastern Ethiopia. Bus. Manag. Econ. Res..

[14] Statistics South Africa (Stassa) Census (2014). Pretoria: Statistics South Africa. www.statssa.gov.za.

[15] Sekhukhune District Municipality Integrated Development Plan (IDP 2015/2016). http://.www.sekhukhunedistrict.gov.za/sdm-admin/doc.

[16] Murovhi N.R., Materechera S.A. (2013). Growth and nutrient uptake by maize (*Zea mays* L.) in a soil amended with leaf litter from sub-tropical fruit trees in Mpumalanga Province, South Africa. J. Food Agric. Environ..

[17] Nell F.J., Siebrits F.K. (1992). Studies on the nutritive value of cowpeas (*Vigna unguiculata*). S. Afr. J. Anim. Sci..

[18] Faber M., Oelofse A., Van Jaarsveld P.J., Wenhold F.A.M., Jansen van Rensburg W.S. (2010). African leafy vegetables consumed by households in the Limpopo and KwaZulu-Natal provinces in South Africa. S. Afr. J. Clin. Nutr..

[19] Seti S. Subsistence gardening for food security: A case study of three townships in Grahamstown, Eastern Cape province. Fort Hare Institute of Social and Economic Research Working Paper No. 53. Presented at Eastern Cape Historical Legacies and New Challenges Conference.

[20] Maanda M.Q., Bhat R.B. (2010). Wild vegetables in the Venda region of Limpopo Province. Int. J. Exp. Bot..

[21] World Bank (2007). World Development Report 2008 Overview: Agriculture for Development.

[22] Rockefeller Foundation (2006). Africa’s Turn: A New Green Revolution for the 21st Century.

[23] Musinguzi E., Kikafunda J.K., Kiremire B. (2003). Utilization of indigenous food plants in Uganda: A case study of South Western Uganda. AJFAND.

[24] Zemedu L., Mesfin W. (2014). Smallholders’ vulnerability to food insecurity and coping strategies: In the face of climate change, East Hararghe, Ethiopia. J. Econ. Sustain. Dev..

[25] Cooper P.J.M., Dimes J., Rao K.P.C., Shapiro B., Shiferaw B., Twomlow S. (2008). Coping better with current climatic variability in the rain-fed farming systems of Sub-Saharan Africa: An essential first step in adapting to future climate change. Agric. Ecosyst. Environ..

[26] Müller C., Cramer W., Hare W.L., Lotze-Campen H. (2011). Climate change risks for African agriculture. Proc. Natl. Acad. Sci. USA.

[27] Rampedi I.T., Olover J. (2013). Traditional beverages derived from wild food plant species in the Vhembe District, Limpopo Province in South Africa. Ecol. Food Nutr..

[28] Baiphethi M.N., Jacobs P.T. (2009). The contribution of subsistence farming to food security in South Africa. Agrekon.

[29] Chadare F.J.D.P., Gayet P., Azakpota M.J.R., Nout A.R., Linneman J., Hounhouigan D., Van Boekel M.A.J.S. (2010). Three traditional fermented baobab foods from Benin, Mutchayan, Dikouanyouri and Tayabounta: Preparation, properties and consumption. Ecol. Food Nutr..

[30] Shumsky S.A., Hickey G.M., Pelletier B., Johns T. (2014). Understanding the contribution of wild edible plants to rural social-ecological resilience in semi-arid Kenya. Ecol. Soc..

[31] Nedelcheva A. (2013). An Ethnobotanical Study of Wild Edible Plants in Bulgaria. Eurasia. J. Biosci..

[32] Kwenin W.K.J., Wolli M., Dzomeku B.M. (2011). Assessing the nutritional value of some African indigenous green Leafy Vegetables in Ghana. J. Anim. Plant Sci..

[33] Modisane P.C., Beletse Y.G., Du Plooy C.P. Yield response of Amaranthus tricolor and Cleome gynandra to fertilizer application. Proceedings of the African Crop Science Conference.

[34] Felhaber T. (1997). South African Primary Health Care Handbook: Combining Western and Traditional Practices.

